# Pediatric Acute Liver Failure Secondary to Autoimmune Hepatitis in an Infant With Thrombocytopenia-Absent Radius (TAR) Syndrome: A Case Report

**DOI:** 10.1097/PG9.0000000000000325

**Published:** 2023-06-09

**Authors:** Rebecca Mercedes, Kalyani Patel, Henry Shiau, Krupa R. Mysore, Wenly Ruan, Daniel H. Leung, Mary Elizabeth M. Tessier, Dana Cerminara, Sarah Nicholas, Kelby Fuller, Marielle Faraone, N. Thao N. Galvan, John Goss, Anna M. Banc-Husu

**Affiliations:** From the * Division of Gastroenterology, Hepatology, and Nutrition, Department of Pediatrics, Baylor College of Medicine, Texas Children’s Hospital, Houston, TX; † Department of Pathology and Immunology, Baylor College of Medicine, Houston, TX; ‡ Division of Gastroenterology, Hepatology, and Nutrition, Department of Pediatrics, The University of Alabama at Birmingham, Children’s of Alabama, Birmingham, AL; § Department of Pharmacy, Texas Children’s Hospital, Houston, TX; ∥ Division of Immunology, Allergy, and Retrovirology, Department of Pediatrics, Baylor College of Medicine, Texas Children’s Hospital, Houston, TX; ¶ Division of Abdominal Transplantation and Hepatobiliary Surgery, Department of Surgery, Baylor College of Medicine, Houston, TX.

**Keywords:** liver transplant, liver-kidney microsomal antibody, antibody-mediated rejection

## Abstract

Thrombocytopenia absent radius (TAR) syndrome is a rare genetic disorder that has been associated with food protein–induced allergic proctocolitis and transient leukemoid reactions, among other manifestations. There has been no prior reports of its association with autoimmune disease, more specifically, autoimmune hepatitis (AIH) or the development of pediatric acute liver failure (PALF). We present a case of an 8-month-old infant with TAR syndrome who presented with PALF, secondary to AIH with elevated liver-kidney microsomal antibody (>1:2560). She received a liver transplant and had a very complicated postoperative course including severe T-cell–mediated rejection, infection, biliary stricture, persistently elevated liver-kidney microsomal antibodies, and antibody-mediated rejection. Ultimately, these complications led to graft failure, severe sepsis, and death. This case highlights a new association of TAR syndrome with AIH and PALF and a potentially aggressive nature of AIH both pre- and post-transplant.

## INTRODUCTION

Thrombocytopenia absent radius (TAR) syndrome is a rare genetic disorder defined by 2 main criteria: hypomegakaryocytic thrombocytopenia typically with platelet count <50 000/µL and the absence of the radii bilaterally with the presence of thumbs (1,2). Additional clinical characteristics include food protein–induced allergic proctocolitis, transient leukemoid reactions, and other skeletal, cardiac, and genitourinary anomalies (1,2). However, autoimmune disease has not been described in TAR. To the best of our knowledge, we describe the first case of pediatric acute liver failure (PALF) requiring liver transplantation (LT) secondary to autoimmune hepatitis (AIH) in a patient with TAR syndrome.

## CASE REPORT

An 8-month-old female with TAR syndrome presented with acute liver failure (ALF). Her TAR syndrome was confirmed on prenatal genetic testing with chromosomal microarray showing paternally inherited deletion of chromosome band 1q21.1 and fetal genetic testing identifying hemizygous RBM8A gene variant (3). There was no reported family history of any liver disease, metabolic disease or autoimmune disorders. She was diagnosed with food protein–induced allergic proctocolitis and required multiple platelet and blood transfusions for hematochezia. She had no known liver disease with normal alanine aminotransferase (ALT) and conjugated bilirubin on routine labs at 1 month old.

She developed acute abdominal distention, irritability, low-grade fever, and scleral icterus at 8 months of age. Initial labs showed ALT 2696 U/L, conjugated bilirubin 3.6 mg/dL, and international normalized ratio 2.2. She had hyperammonemia (81 µmol/L) but no evidence of hypoglycemia. Platelets were 26 × 10^3^/µL with last platelet transfusion 9 days before presentation. Right upper quadrant ultrasound showed diffusely heterogeneous liver with background starry sky appearance and patent vasculature.

**FIGURE 1.**
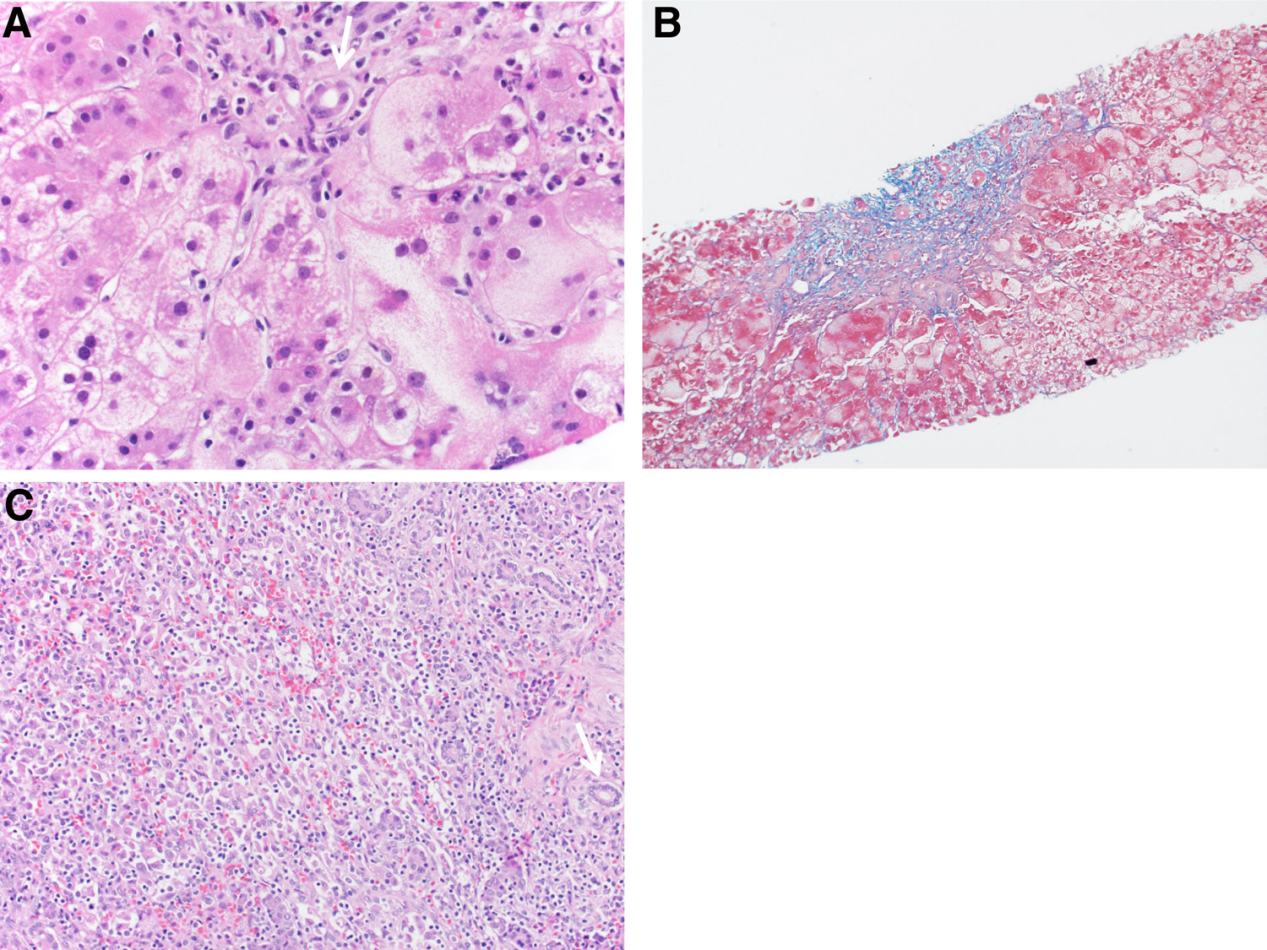
Liver biopsy and explant specimens of native liver. A) Native liver biopsy at 8 mo of age showing giant cell hepatitis. (H&E, 400×) B) Trichrome stain shows minimal portal fibrous expansion. (100×) C) Explant liver showed extensive parenchymal loss with severe lymphohistiocytic inflammation. Bile duct is highlighted by an arrow in (A) and (C).

Liver biopsy on hospital day (HD) 5 showed neonatal giant cell hepatitis, absent/minimal fibrosis (Fig. 1A, B) without features of distal biliary obstruction or lymphoplasmacytic hepatitis. An extensive evaluation was completed for metabolic, infectious, and autoimmune etiologies of PALF and was negative except for a positive anti-liver-kidney microsomal-1 antibody (LKM) of >1:2560, which resulted after LT (Supplemental Digital Content, Table 1, http://links.lww.com/PG9/A114). As pre-LT liver biopsy was not consistent with AIH and antibody titers had not resulted, treatment with steroids was not pursued. On HD8, she developed progressive hepatic encephalopathy, worsening cholestasis, and coagulopathy and was listed status 1A for LT. On HD10, she underwent an uncomplicated ABO-compatible, whole organ LT with choledochocholedochostomy anastomosis. Explant liver showed extensive parenchymal loss with mixed lymphohistiocytic and rare plasmacytic inflammation (Fig. 1C).

**FIGURE 2.**
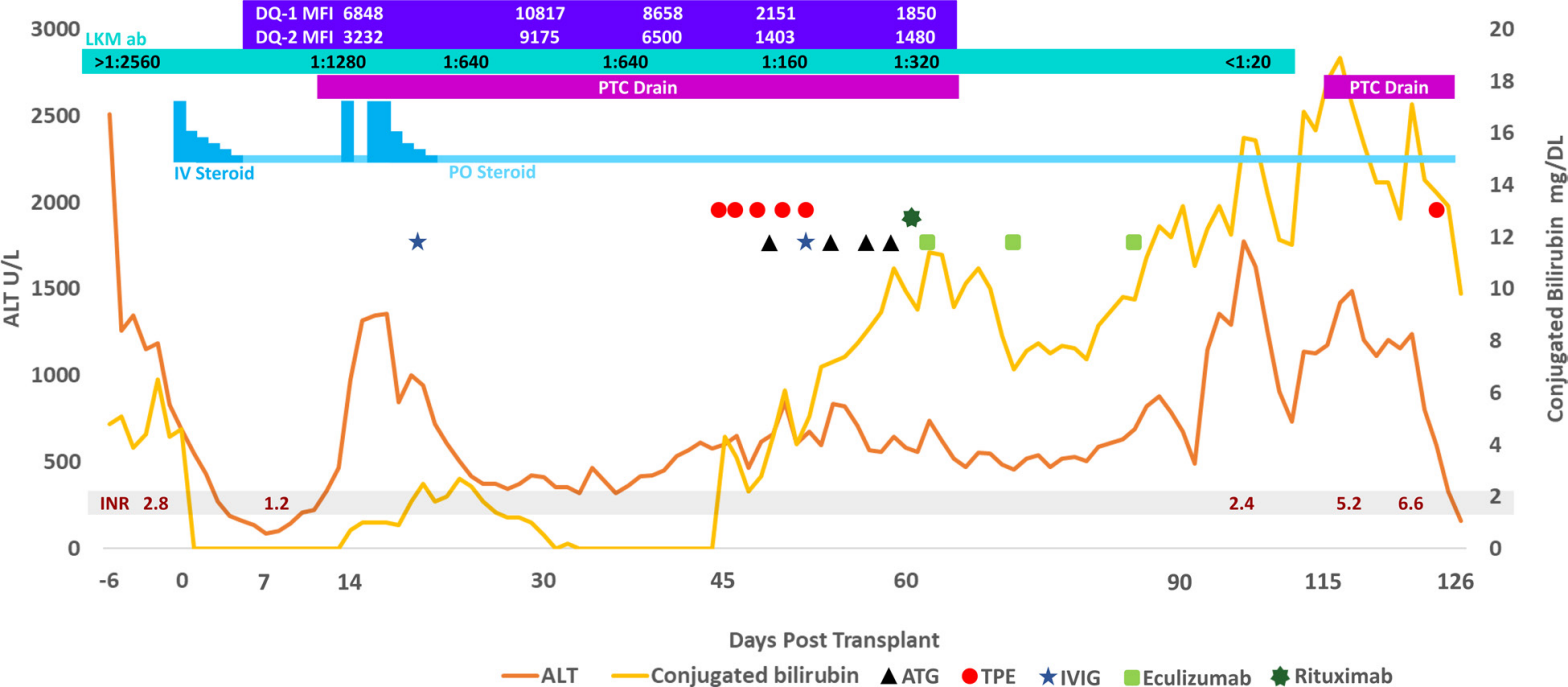
Pre/post-transplant laboratory trends and intervention timeline. ALT = alanine aminotransferase; ATG = anti-thymocyte globulin; DQ-1 MFI = DQ mean fluorescence intensity for donor-specific antibody; DQ-2 MFI = DQ mean fluorescence intensity for donor-specific antibody; IV = intravenous; IVIG = intravenous immunoglobulin; LKM ab = liver-kidney microsomal antibody; PO = per os; PTC drain = percutaneous transhepatic cholangiogram drain; TPE = therapeutic plasma exchange.

**FIGURE 3.**
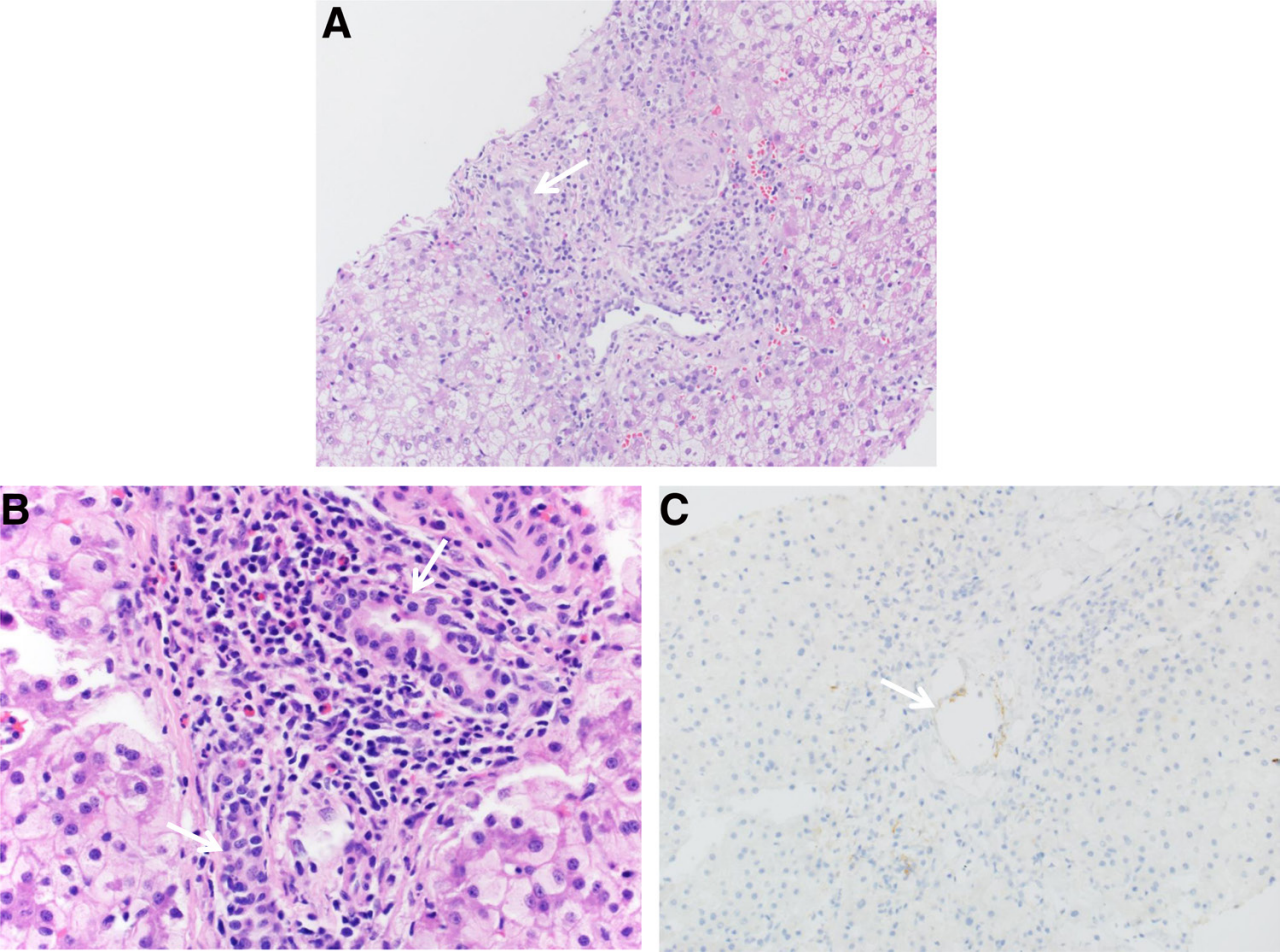
Liver biopsy specimens post-transplant showing multiple complications including severe rejection and antibbody-medicated rejection. A) Allograft biopsy POD12 showed severe acute cellular rejection. (H&E, 200×) B) Allograft biopsy POD44 continued to show severe acute cellular rejection with mixed portal inflammation along with eosinophils. (H&E, 400×). C) Immunohistochemical stain for C4d from the POD44 biopsy shows rare positive staining in the peri-biliary capillaries (arrow). (200×) Arrows in (A) and (B) highlight bile ducts. POD = postoperative day.

She received induction immunosuppression with intravenous (IV) methylprednisolone, maintenance immunosuppression with steroids and tacrolimus, and standard infection prophylaxis (trimethoprim/sulfamethoxazole, fluconazole, and ganciclovir). On postoperative day (POD) 4, mycophenolate was added once LKM antibody and explant pathology resulted (simplified AIH score of 6) due to concerns for possible AIH (4). Post-LT course was complicated by human herpes virus 6 (HHV-6) viremia, biliary stricture, steroid refractory T-cell mediated rejection (TCMR), and antibody-mediated rejection (AMR) (Fig. 2). On POD12, liver biopsy performed for elevated liver enzymes showed features of severe TCMR with negative C4d staining (Fig. 3A). Repeat biopsies in the setting of persistently elevated liver transaminases were concerning for steroid refractory TCMR (Fig. 3B) and AMR with persistently positive and increasing C4d staining (Fig. 3C). She had donor-specific antibodies measured during her course with rising titers over time and persistently elevated LKM antibody, and on POD45, she began treatment for AMR (Fig. 2).

Despite these interventions, she developed worsening graft failure with rising conjugated bilirubin and coagulopathy and was relisted for LT 5 months after her initial presentation. She experienced recurrent cholangitis which progressed to disseminated carbapenem-resistant Enterobacter cloacae infection. She eventually developed overwhelming sepsis and subsequent cerebral edema with herniation and died prior to a second LT.

## DISCUSSION

TAR syndrome is a rare clinical syndrome with no previously reported cases of AIH or ALF requiring LT. There are many documented hematologic manifestations associated with TAR syndrome; most commonly transient leukemoid reaction with white blood cell count >35 000/µL and often associated with hepatosplenomegaly (1). Other rare reports include T-cell acute lymphoblastic leukemia (ALL), B-cell ALL, acute myeloid leukemia, and Langerhans cell histiosyctosis (5,6). While there are no reports of autoimmune disease or AIH in patients with TAR syndrome, TAR is very rare and it is possible that cases exist that have not been reported or that this is rather just a casual association rather than TAR leading to increased susceptibility to AIH. At the time of LT in our patient, the etiology of her ALF was unknown. Her initial liver biopsy showed findings of giant cell hepatitis without clear features of AIH. Her significantly elevated pre-transplant LKM antibody level resulted post-LT and it was initially unclear if this was secondary to circulating maternal antibodies given her age as well as extremely high titer. Post-transplant, the patient’s mother was found to have negative LKM antibody and the patient’s remained elevated although IgG was normal, supporting the pre-LT diagnosis of AIH in the patient. Her simplified AIH score of 6 and explant pathology showing lymphoplasmacytic infiltration suggests probable AIH, although accuracy of scoring systems in the setting of ALF may be limited (7). In a large pediatric AIH study, ALF was found to occur more frequently in type II (LKM antibody positivity) than type I patients (25% versus 3%) (7–9). Overall, AIH accounts for approximately 2%–5% of the pediatric LTs in the United States and Europe and of only 21% of patients with AIH requiring LT were for ALF (8,10). It is unclear if this patient’s underlying TAR predisposed her to a severe presentation of AIH with ALF, especially in light of other bone marrow manifestations seen in TAR. It is important to consider possible autoimmune diseases, especially AIH, with TAR that has associated elevated liver enzymes. Her complex post-LT course also may suggest a component of immune dysregulation in patients with TAR, especially given the severity of TCMR and the development of AMR that was difficult to treat.

One of the other challenging aspects of this case was the presence of multiple competing diagnoses contributing to her ongoing graft dysfunction. Although AMR is thought to be much less prevalent in LT, the diagnosis of AMR for this patient was challenging. One study demonstrated an increased rate of HLA antibodies in AIH, which may indicate a possible mechanism of liver injury in AIH as well as higher risk of acute AMR post-transplant in these patients, though further studies are needed (11). Additionally, in cardiac transplantation, transfusion of blood products increases anti-HLA antibodies and risk of development of AMR post-transplant due to increased antigenic exposure (12). Our patient received multiple blood and platelet transfusions throughout her life due to her underlying TAR, which also may have potentially played an important role in her clinical course.

In addition to the many complexities in this case, the underlying diagnosis of TAR syndrome with significant thrombocytopenia posed an additional challenge in her management, especially surrounding procedures and coordination of platelet transfusions to prevent hemorrhage both before and after transplant. While it remains unclear if there is a causal relationship between her underlying TAR syndrome and development of ALF secondary to AIH, this unique case presents important considerations for all physicians caring for children with TAR in the setting of elevated transaminases. Early screening for AIH may be warranted in such patients and further understanding of the development of autoimmunity in patients with TAR is needed.

## ACKNOWLEDGMENTS

The authors would like to thank the patient’s parents/guardians who provided informed consent for publication of this case report.

## Supplementary Material
