# Supplementary material for: Pediatric Acute Liver Failure Secondary to Autoimmune Hepatitis in an Infant With Thrombocytopenia-Absent Radius (TAR) Syndrome: A Case Report
Source: JPGN Rep. 2023 Jun 9;4(3):e325. doi: 10.1097/PG9.0000000000000325 (PMC10435028; doi:10.1097/PG9.0000000000000325)
Supplement: Supplementary file 1 [file pg9-4-e325-s001.pdf]

Table 1: Acute liver failure laboratory evaluation

Test	Result
**Infectious**	
Blood culture	No growth
Urine culture	No growth
Respiratory viral panel	Negative
GI pathogen panel	Negative
HIV	Negative
Viral hepatitis	
Hepatitis A antibody	Positive
Hepatitis A IgM	Negative
Hepatitis B surface antigen	Negative
Hepatitis B core antibody	Negative
Hepatitis C antibody	Negative
RPR	Non-reactive
CMV	
IgG	Negative
IgM	Negative
PCR	Negative
EBV	
IgM	Negative
IgG	Positive
HSV 1	
IgG	Positive
PCR	Negative
HSV 2	
IgG	Negative
PCR	Negative
Adenovirus PCR	Negative
Enterovirus PCR	Negative
HHV6 PCR	Negative
Parvovirus B19	Negative
**Autoimmune**	
ANA profile	FANA titer 1:320 (elevated)
Liver Kidney microsomal antibody	>1:2560 (elevated)
IgG	1030 (normal)
F actin IgG	19 (normal)
**Metabolic**	
Urine organic acids	Normal
Plasma amino acids	
Methionine	718 (normal 9-45)
tyrosine	333 (normal 20-96)
Urine Succinylacetone	Normal
Acylcarnitine profile	Normal

Homocysteine	11 (normal 3.3-8.3)
Pyruvate dehydrogenase complex	Normal
**Miscellaneous**	
Thyroid stimulating hormone	Normal
Free T4	Normal
Alpha 1 antitrypsin genotype	MS type
Ceruloplasmin	Normal
Ferritin	124 (normal 10-95)
Alpha fetal protein	134
